# Crystal structure of a GCN5-related *N*-acetyltransferase from *Lactobacillus curiae*

**DOI:** 10.1107/S2053230X2300571X

**Published:** 2023-08-09

**Authors:** Jennifer R. Fleming, Franziskus Hauth, Jörg S. Hartig, Olga Mayans

**Affiliations:** a Department of Biology, University of Konstanz, Universitätsstrasse 10, 78457 Konstanz, Germany; b Department of Chemistry, University of Konstanz, Universitätsstrasse 10, 78457 Konstanz, Germany; c Konstanz Research School Chemical Biology (KoRS-CB), University of Konstanz, Universitätsstrasse 10, 78457 Konstanz, Germany; Centre for Cellular and Molecular Biology, Hyderabad, India

**Keywords:** GCN5-related *N*-acetyltransferases, *Lacto­bacillus curiae*, canavanine, guanidine riboswitch-associated gene functions, X-ray crystallography, acetylation

## Abstract

Members of the GCN5-related *N*-acetyltransferase (GNAT) family are found in all domains of life and are involved in processes ranging from protein synthesis and gene expression to detoxification and virulence. Due to the variety of their macromolecular targets, GNATs are a highly diverse family of proteins. Currently, 3D structures of only a small number of GNAT representatives are available and thus the family remains poorly characterized. Here, the crystal structure of the guanidine riboswitch-associated GNAT from *Lacto­bacillus curiae* (*Lc*GNAT) that acetylates canavanine, a structural analogue of arginine with antimetabolite properties, is reported. *Lc*GNAT shares the conserved fold of the members of the GNAT superfamily, but does not contain an N-terminal β0 strand and instead contains a C-terminal β7 strand. Its P-loop, which coordinates the pyrophosphate moiety of the acetyl-coenzyme A cosubstrate, is degenerated. These features are shared with its closest homologues in the polyamine acetyltransferase subclass. Site-directed mutagenesis revealed a central role of the conserved residue Tyr142 in catalysis, as well as the semi-conserved Tyr97 and Glu92, suggesting that despite its individual substrate specificity *Lc*GNAT performs the classical reaction mechanism of this family.

## Abstract 2

The 3D structure of a GCN5-related *N*-acetyltransferase enzyme that is selective for canavanine has been elucidated and shown to share the fold and catalytic mechanism of the polyamine acetyltransferase subclass.

## 1. Introduction

Acetylation is a major post-translational modification that is found in all domains of life (Favrot *et al.*, 2016 ▸). It was first described as a regulatory mechanism in the 1960s with the discovery of histone acetylation (Phillips, 1963 ▸) and the discovery of a bacterial aminoglycoside acetyltransferase, which was shown to confer antibiotic resistance (Okamoto & Suzuki, 1965 ▸). The importance of this ubiquitous modification has become progressively established in the past decades and it is now known to occur in multiple molecular targets, including proteins, polyamines, toxins, transfer RNA and cell-wall components (Burckhardt & Escalante-Semerena, 2020 ▸). Accordingly, it has widespread involvement in many cellular processes. Acetylation is catalysed by acetyltransferases, which represent one of the largest known protein superfamilies, with more than 300 000 representatives (Salah Ud-Din *et al.*, 2016 ▸). Acetyltransferases can be divided into three main classes: MYST[MOU1] (Pfam01853), p300/CBP[MOU2] (Pfam06466) and GCN5-related *N*-acetyltransferases (GNATs; Pfam00583) (Burckhardt & Escalante-Semerena, 2020 ▸). Despite their sequence and structural diversity, all acetyltransferases function by transferring an acetyl group from the cosubstrate acetyl-coenzyme A (Ac-CoA) to the amino group of a specific substrate; the substrates can be very diverse across the enzymes, ranging from small metabolites such as amino acids to secondary metabolites such as antibiotics. In the GNAT class, the acetyl group can be transferred to either the ɛ-amino group (N^ɛ^) of a lysine residue or the α-amino group (N^α^) of an N-terminal residue (Favrot *et al.*, 2016 ▸).

Prokaryotes only possess the GNAT class of acetyltransferases (Hentchel & Escalante-Semerena, 2015 ▸). GNATs catalyse a common modification but share little sequence homology (3–23%; Vetting *et al.*, 2005 ▸). Recently, a group of putative GNAT enzymes have been shown to be guanidine riboswitch-associated (Lenkeit *et al.*, 2020 ▸; Salvail *et al.*, 2020 ▸). Riboswitches are small regulatory RNAs that can regulate gene expression upon specifically binding a given ligand (guanidine in the case of the GNATs). Interestingly, a representative of such guanidine riboswitch-associated GNATs, a GNAT from *Lactobacillus curia* (*Lc*GNAT), performs acetyl­ation of the arginine antimetabolite canavanine (or δ-oxa-arginine) but not of arginine itself (Lenkeit *et al.*, 2023 ▸). *Lc*GNAT is the only canavanine-acylating GNAT identified to date. It was further found that canavanyl-tRNA^Arg^ deacylase (CtdA) is also guanidine riboswitch-associated (Hauth *et al.*, 2023 ▸), and both *Lc*GNAT and CtdA are found in the same biological habitats, specifically canavanine-rich habitats such as the legume rhizosphere or herbivore gut. Hence, it has been suggested that *Lc*GNAT serves a similar biological purpose as CtdAs, namely prevention of the misincorporation of canavanine into the bacterial proteome, as acetylation makes canavanine unusable for protein synthesis by the ribosome.

Here, we resolved the 3D structure of the guanidine riboswitch-associated enzyme *Lc*GNAT to gain an insight into the mechanistic diversity of this protein family and to facilitate future research towards revealing its catalytic mechanism. Specifically, gaining insight into the discrimination between the closely related canavanine and arginine substrates is a future goal.

## 2. Methods

### 2.1. Cloning

The full-length, codon-optimized gene for *Lc*GNAT (NCBI WP_035166819.1) was commercially synthesized (ThermoFisher) and cloned into the expression vector pET-28a (EMBL vector collection) by restriction-site cloning and quick ligation (NEB). The vector adds an N-terminal His_6_ tag and a Tobacco etch virus (TEV) protease-cleavage sequence N-terminal to the inserted gene of interest.

*Lc*GNAT variants carrying a single amino-acid mutation were generated by whole-plasmid overhang PCR followed by quick ligation (NEB).

All resulting clones were verified by sequencing (GATC, Eurofins).

### 2.2. Protein production

For protein expression, expression plasmids were transformed into *Escherichia coli* BL21(DE3) strain Gold (Agilent) and a starter culture was grown overnight in Luria–Bertani medium supplemented with kanamycin (30 µg ml^−1^) at 37°C. After a 1:500 dilution, the culture was further cultivated at 37°C and 200 rev min^−1^ to an OD_600_ of approximately 0.5. Protein expression was then induced with 0.5 m*M* isopropyl β-d-1-thiogalactopyranoside (IPTG) and the culture was further grown at 18°C for approximately 16 h. The cells were harvested by centrifugation and stored at −20°C. Subsequently, the cells were resuspended in 50 m*M* Tris–HCl pH 8.0, 100 m*M* NaCl, 20 m*M* imidazole containing protease-inhibitor cocktail (cOmplete Mini, EDTA-free, Merck) and lysed by sonication. The sample was purified by immobilized metal-affinity chromatography (IMAC) using Ni^2+^–NTA agarose (Qiagen) and was eluted with 50 m*M* Tris–HCl pH 8.0, 100 m*M* NaCl, 500 m*M* imidazole. This was followed by removal of the His_6_ tag by the addition of TEV protease. The sample was then dialyzed against 50 m*M* Tris–HCl pH 8.0, 100 m*M* NaCl to remove excess imidazole and subtractive IMAC was performed using Ni^2+^–NTA agarose (Qiagen) to remove the protease, the cleaved tag and any *Lc*GNAT that retained a tag. To perform size-exclusion chromatography, the sample (with an approximate volume of 3 ml) concentrated to 6 mg ml^−1^ was loaded onto a Superdex S75 16/60 column (GE Healthcare) pre-equilibrated in 50 m*M* Tris pH 8.0, 100 m*M* NaCl. The resulting protein sample was estimated to be >95% pure using SDS–PAGE stained with Coomassie Blue. Finally, the sample was concentrated to 15 mg ml^−1^ and stored at 4°C until further use. The approximate yield of purified *Lc*GNAT was 8 mg per litre of *E. coli* culture. Protein concentrations were determined from *A*_280_ values measured using a UV–Vis spectrophotometer (Eppendorf) by applying the Beer–Lambert law using a molar extinction coefficient (ɛ = 23 950 *M* ^−1^ cm^−1^ at 280 nm) calculated from the sequence data using *ProtParam* (Gasteiger *et al.*, 2005 ▸).

### 2.3. X-ray crystallography

Crystals of *Lc*GNAT grew from solutions consisting of 1 *M* potassium sodium tartrate, 0.1 *M* MES–NaOH pH 6.0 in Intelli-Plates (Art Robbins) using the sitting-drop vapour-diffusion method at 18°C. Crystallization drops consisted of a 1:1 ratio of protein solution and reservoir solution and had a volume ratio of 200:200 nl. For X-ray irradiation, crystals were cryoprotected with Paratone-N (Hampton Research) prior to flash-vitrification in liquid nitrogen.

X-ray diffraction data were collected on beamline PXI at the Swiss Light Source (SLS) synchrotron, Villigen, Switzerland under cryo-conditions (100 K). Data processing used *XDS* and *XSCALE* (Kabsch, 2010 ▸). Phasing was performed by molecular replacement in *Phaser* (McCoy *et al.*, 2007 ▸) using a putative acetyltransferase from *Streptococcus mutans* (PDB entry 4e2a; sequence identity 38.6%; G.-L. Li, J.-K. Nie, L.-F. Li & X.-D. Su, unpublished work) pruned to common atoms with *Sculptor* (Bunkóczi & Read, 2011 ▸) as a search model. Manual model building was performed in *Coot* (Emsley *et al.*, 2010 ▸) and model refinement was carried out in *phenix.refine* (Liebschner *et al.*, 2019 ▸) using isotropic *B* factors and TLS parameters (one group per polypeptide chain in the asymmetric unit). The quality of the final model was assessed using *MolProbity* (Williams *et al.*, 2018 ▸). Molecular images were rendered using *UCSF Chimera* (Pettersen *et al.*, 2004 ▸). X-ray data statistics and model parameters are given in Table 1 ▸.

### 2.4. Acetylation activity assay

Acetylation activity was determined using Ellman’s reagent (5,5′-dithiobis-2-nitrobenzoic acid; DTNB) as described previously (Lenkeit *et al.*, 2023 ▸). In brief, 5 µ*M* purified protein (wild type or point mutated) was incubated with 20 µl reaction mixture (20 m*M* Tris–HCl pH 7.5, 200 m*M* NaCl, 0.5 m*M* acetyl-CoA, 1.25 m*M* canavanine) in a 96-well plate. After incubation for 5 min at 28°C, 25 µl stop buffer (100 m*M* Tris–HCl pH 8.0, 8 *M* urea) was added, followed by the addition of 100 µl DTNB reagent (100 m*M* Tris–HCl pH 8.0, 2 m*M* DTNB, 1 m*M* EDTA). After 5 min of incubation at room temperature, the *A*_420_ was measured. For quantification, different concentrations of CoA were used to obtain a calibration curve.

### 2.5. Bioinformatics

To identify structural homologues of *Lc*GNAT with known 3D structure, its structure was used in a search of the Protein Data Bank (PDB; https://www.rcsb.org) using the *DALI* server (https://ekhidna2.biocenter.helsinki.fi/dali) as a search engine. Structures with a sequence conservation of >20% that contained Ac-CoA or CoA as ligands were selected, namely *Bacillus subtilis* PaiA (*Bs*PaiA; PDB entry 1tiq), *Streptococcus mutans* putative acetyltransferase (*Sm*GNAT; PDB entry 4e2a), *Thermoplasma acidophilum* PaiA (*Ta*PaiA; PDB entry 3k9u) and *T. volcanium* *N*-acetyltransferase (*Tv*Ard1; PDB entry 4pv6). Their sequences were then aligned using *Clustal Omega* (Sievers *et al.*, 2011 ▸).

Highly conserved residues in *Lc*GNAT were identified using the *ConSurf* web server (https://consurf.tau.ac.il/consurf_index.php) and its top 150 closest homologues. Residues involved in Ac-CoA interactions were determined using the annotation in the sequence inspector in the PDB (Berman *et al.*, 2000 ▸) and were confirmed through visual inspection of 3D structures in *UCSF Chimera* (Pettersen *et al.*, 2004 ▸). Pairwise sequence alignments were conducted using the *EMBOSS Needle* web server (https://www.ebi.ac.uk/Tools/psa/emboss_needle/). The significance of intermolecular inter­actions was assessed using *PISA* (https://www.ebi.ac.uk/pdbe/prot_int/pistart.html; Krissinel & Henrick, 2007 ▸).

## 3. Results and discussion

### 3.1. *Lc*GNAT has a conserved fold

**Figure 1:**
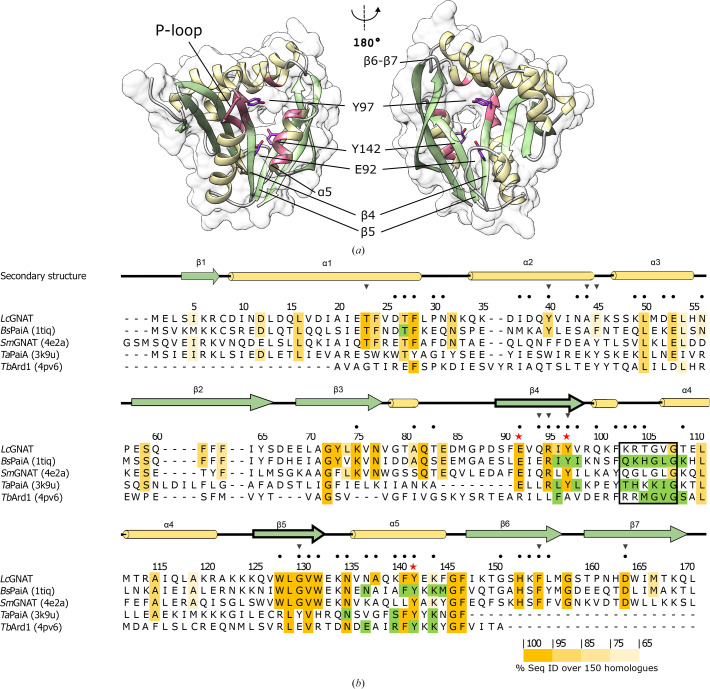
The structure and sequence of *Lc*GNAT is conserved among polyamine acetyltransferases. (*a*) Crystal structure of *Lc*GNAT. Catalytic residues that are subjected to mutagenesis in this study are displayed. The P-loop is also highlighted. (*b*) Sequence alignment of *Lc*GNAT and its four closest homologs with known 3D structures. The sequences correspond to *Bacillus subtilis* PaiA (*Bs*PaiA), *Streptococcus mutans* putative acetyltransferase (*Sm*GNAT), *Thermoplasma acidophilum* PaiA (*Ta*PaiA) and *T. volcanium* *N*-acetyltransferase (*Tv*Ard1). PDB codes are given in parentheses. Residue numbering uses the *Lc*GNAT sequence. Secondary structure is colour-coded as in (*a*), where β-strands are shown in green and α-­helices are in yellow. Heavy outlined β-strands indicate the two strands involved in the formation of the V-shaped splay. Sequence identity is shown in yellow. Ac-CoA-binding residues were defined by PDB sequence annotations and are shown in green. The P-loop is boxed. Black dots indicate residues that are part of the substrate tunnel. Red stars highlight residues that were mutated in the acetylation activity assay.

The atomic structure of *Lc*GNAT has been elucidated to 1.95 Å resolution using X-ray crystallography (Fig. 1 ▸, Table 1 ▸). Representative electron density is shown in Supplementary Fig. S1. The crystal form obtained in this study contains two molecular copies in the asymmetric unit, which are essentially identical (r.m.s.d. of 0.73 Å for all 174 C^α^ atoms). The exclusion volume of *Lc*GNAT in size-exclusion chromatography suggested the presence of a single species with approximate molecular mass 23 kDa (Supplementary Fig. S2). As the molecular mass of *Lc*GNAT calculated from the sequence data is 20.25 kDa, this indicates that the enzyme is monomeric in solution under the experimental conditions used. An inspection of the protein–protein interface of the dimer in the crystallographic asymmetric unit, as well as potential dimers across crystallographic axes, using *PISA* assigned the lowest complex-formation significance score of 0 to the resulting molecular interfaces, suggesting that molecular interfaces in the crystal are the result of lattice packing only. Thus, we conclude that *Lc*GNAT is likely to be monomeric in its biologically relevant state.

The *Lc*GNAT fold approximates, but does not exactly follow, the canonical topology of the GNAT enzyme family: β0–β1–α1–α2–β2–β3–β4–α3–β5–α4–β6 (Favrot *et al.*, 2016 ▸; Burckhardt & Escalante-Semerena, 2020 ▸). Instead, *Lc*GNAT consists of seven β-strands and four α-helices with composition β1–α1–α2–β2–β3–β4–α3–β5–α4–β6–β7, therefore lacking an N-terminal β0 strand and including a C-terminal β7 strand (Fig. 1 ▸ *a* and Supplementary Fig. S3). This fold divergence has been observed in various other polyamine acetyltransferases (see below) and agrees with the known fact that the N- and C-termini of this fold contain the least conserved secondary-structure elements (Salah Ud-Din *et al.*, 2016 ▸). As is also common to members of this enzyme family, *Lc*GNAT contains an extensive tunnel which perforates the protein fold and is generated by a V-shaped splaying of β-strands β4 and β5 (Fig. 1 ▸ *a*). This V-shaped feature accommodates the pantothenate moiety of Ac-CoA in GNATs (Wybenga-Groot *et al.*, 1999 ▸) and has also been reported to be involved in the formation of the oxyanion hole that polarizes the thioester carbonyl reaction intermediate (Bhatnagar *et al.*, 1998 ▸; Farazi *et al.*, 2001 ▸). At one entrance to the tunnel is the pyrophos­phate-binding loop (P-loop; Fig. 1 ▸ *a*), which binds the pyrophosphate moiety of the Ac-CoA cosubstrate. The sequence of the P-loop is highly conserved in GNATs, hosting a consensus motif (R/Q-*X*-*X*-G-*X*-A/G; Favrot *et al.*, 2016 ▸; Burckhardt & Escalante-Semerena, 2020 ▸). Interestingly, the P-loop of *Lc*GNAT is degenerated, as is also often the case in polyamine acetyltransferases (Fig. 1 ▸ *b*, boxed residues). Since the P-loop binds the Ac-CoA cosubstrate (and not the specific target substrate) and all GNATs, including *Lc*GNAT, are Ac-CoA-dependent, it can be inferred that the differences in the sequence in the P-loop do not result in a significant functional difference in these enzymes.

### 3.2. The functional groups of *Lc*GNAT

**Figure 2:**
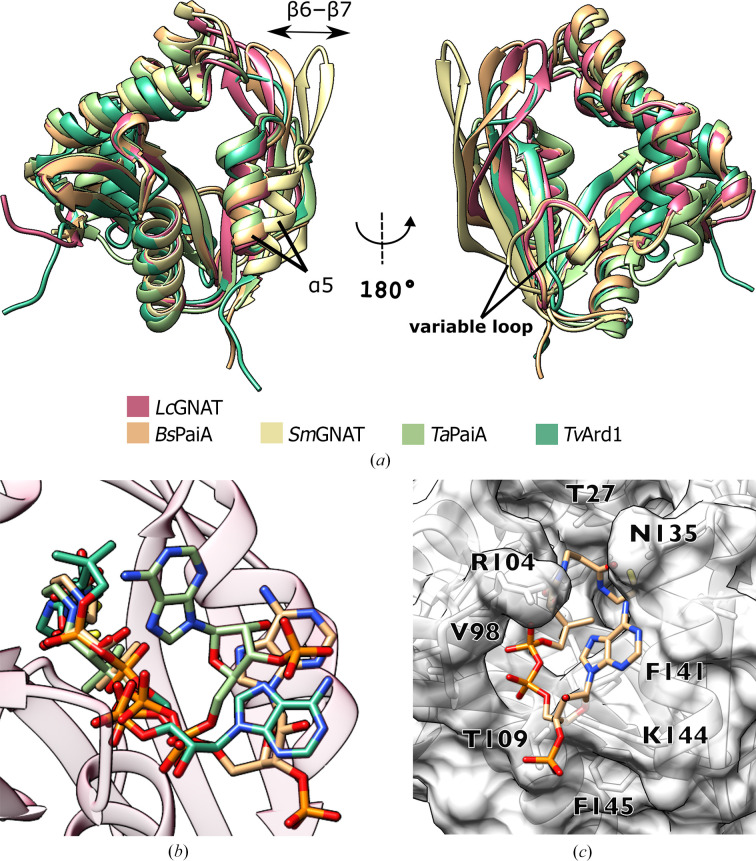
*Lc*GNAT homologues and their cosubstrate-binding modes. (*a*) Structural superimposition of *Lc*GNAT with *Bs*PaiA (PDB entry 1tiq), *Sm*GNAT (PDB entry 4e2a), *Ta*PaiA (PDB entry 3k9u) and *Tv*Ard1 (PDB entry 4pv6). (*b*) Crystal structure of *Lc*GNAT displayed with the Ac-CoA or CoA ligands bound to homologues structurally aligned as in (*a*). Ligands are colour-coded: C atoms correspond to the ribbon colour in (*a*), N atoms are blue, O atoms are red, S is in yellow and P is in orange. (*c*) *Lc*GNAT in surface representation displaying residues which correspond to Ac-CoA-binding residues in *Bs*PaiA (PDB entry 1tiq). The Ac-CoA ligand in *Bs*PaiA is shown in stick representation.

Efforts to elucidate the structure of *Lc*GNAT in complex with Ac-CoA and/or the canavanine substrate in this study were not successful. Thus, we aimed to identify the active-site residues by comparison with characterized homologues of known three-dimensional structure bound to the Ac-CoA cosubstrate. For this, we performed a homology search of the Protein Data Bank. Four close homologues were identified in this way. The closest structural homologue to *Lc*GNAT was a member of the spermidine/spermine-*N* ^1^-acetyltransferase (SSAT) family from the Gram-positive bacterium *Bacillus subtilis* (*Bs*PaiA; PDB entry 1tiq; Forouhar *et al.*, 2005 ▸). Despite its different target substrate, this enzyme shares 46.6% sequence identity and 66.1% sequence similarity with *Lc*GNAT. The next closest homologues were GNAT from *Streptococcus mutants* (*Sm*GNAT; PDB entry 4e2a) and PaiA from *Termoplasma acidophilum* (*Ta*PaiA; PDB entry 3k9u; Filippova *et al.*, 2011 ▸), with sequence identities of 37.0% and 21.7% and similarities of 61.3% and 46.9%, respectively, and the more distantly related Ard1 from *T. volcanium* (*Tv*Ard1; PDB entry 4pv6; Ma *et al.*, 2014 ▸), with a sequence identity of 24.3% and a similarity of 39.1% (Fig. 1 ▸ *b*). As expected from the sequence similarity, *Lc*GNAT and the four identified homologues share the same, closely superimposable fold (Fig. 2 ▸ *a*). Structural variability affects β-hairpin β6–β7, and also helix α5 in *Sm*GNAT, which precedes this β-hairpin. Thus, we concluded that the α5–β6–β7 region is the most flexible and dynamic in this modified version of the GNAT fold.

The structures of *Bs*PaiA and *Tv*Ard1 were elucidated in complex with CoA and those of *Ta*PaiA and *Tv*Ard1 were elucidated in complex with Ac-CoA. In all cases, the co­substrate binds within the tunnel at the centre of the fold. However, despite the close similarity of the shared fold, the binding modes of CoA/Ac-CoA across the different enzymes are remarkably divergent (Fig. 2 ▸ *b*). The binding of the pyrophosphate moiety by the P-loop of the enzyme is the best-shared characteristic across the available liganded structures, with little conservation of cosubstrate conformation existing outside this loop (Fig. 2 ▸ *b*). This conformational diversity does not make it possible to reliably model the complexation of Ac-CoA by *Lc*GNAT. However, given the high sequence similarity of *Lc*GNAT and *Bs*PaiA and the close structural overlap of their structures (r.m.s.d. of 1.27 Å on all 170 C^α^ atoms; Fig. 2 ▸ *a*), we could confirm that the binding mode of Ac-CoA observed in *Bs*PaiA is compatible with *Lc*GNAT (Fig. 2 ▸ *c*). The Ac-CoA thus modelled in *Lc*GNAT was oriented in such a way that the P-loop accommodated the pyrophosphate moiety well and the acetyl group pointed into the tunnel. Unfortunately, efforts to identify the binding site for canavanine in *Lc*GNAT in order to determine how this enzyme achieves its selectivity were unsuccessful. We tried to examine the non-AcCoA side of the tunnel in order to identify the binding interface for canavanine. In particular, we examined residues within a 5 Å distance of the lysine substrate in the recent crystal structure of the distant homologue moss spermine/spermidine acetyltransferase (*Pp*SSAT; PDB entry 7zkt; Bělíček *et al.*, 2023 ▸; Fig. 1 ▸ *b*). Remarkably, the residues thus identified are largely conserved across polyamine acetyltransferases and do not explain the selectivity of *Lc*GNAT for canavanine. This suggests that other residues that are not identifiable at present must also mediate the binding of the larger canavanine substrate in *Lc*GNAT.

### 3.3. Catalytic residues

**Figure 3:**
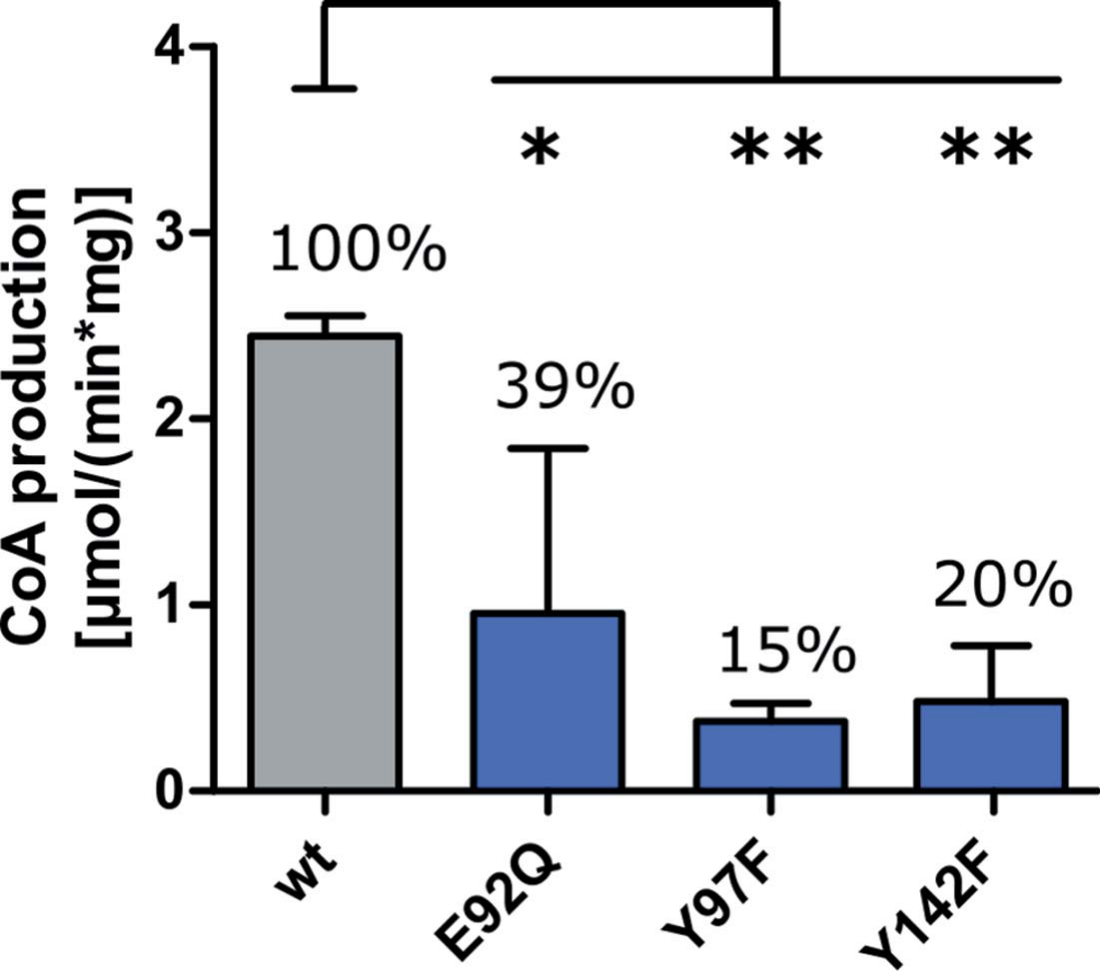
Effect of point mutations on the acetylation activity of *Lc*GNAT. Significance was assessed using *GraphPad Prism* 5.0 by one-way ANOVA followed by Tukey’s multiple comparison test. *, *p* ≤ 0.05; **, *p* ≤ 0.01 (*n* = 3).

An overall sequence alignment of *Lc*GNAT with the four identified homologues described above showed that the enzymes share high conservation in the regions involved in Ac-CoA binding and in residues that are thought to have a catalytic impact (Fig. 1 ▸ *b*). In *Bs*PaiA, the side chain of a conserved tyrosine residue (Tyr142) was shown to interact with the S atom of CoA and has been proposed to serve as a general acid in catalysis (Forouhar *et al.*, 2005 ▸). However, an alternative study of *Enterococcus faecium* GNAT proposed that Tyr147 (equivalent to Tyr142) is not involved in the chemical catalysis of the reaction but instead dictates the optimal orientation of the acetyl group for transfer (Draker & Wright, 2004 ▸). Even though its exact mechanistic role is unclear, the review by Salah Ud-Din *et al.* (2016 ▸) reported that an equivalently positioned tyrosine residue is crucial for catalysis in nearly all GNAT enzymes described to date. In addition, in human SSAT the conserved residue Glu92 was proposed to serve as a general base that performs a water-mediated proton extraction from the substrate (Hegde *et al.*, 2007 ▸). Mutagenesis also confirmed a role of this residue in catalysis in other GNATs (reviewed by Salah Ud-Din *et al.*, 2016 ▸). Both Glu92 and Tyr142 are also present in *Lc*GNAT. Using site-directed mutagenesis, we generated the *Lc*GNAT variants E92Q and Y142F and tested their enzymatic activity, confirming that both residues also impair catalysis in *Lc*GNAT and therefore are catalytically relevant (Fig. 3 ▸). Interestingly, *Lc*GNAT and *Bs*PaiA share an additional tyrosine (Tyr97) in the Ac-CoA binding site that was annotated to mediate cosubstrate binding in *Bs*PaiA (Forouhar *et al.*, 2005 ▸). Both Tyr97 and Tyr142 are at a similar distance from the carbonyl moiety of the acetyl group in the crystal structure of *Bs*PaiA. A similar residue, Tyr93, has also been proposed to be involved in cosubstrate positioning in *Ta*PaiA (Filippova *et al.*, 2011 ▸). However, a tyrosine residue is not conserved in this position across all GNATs (Fig. 1 ▸ *b*). Here, we mutated Tyr97 in *Lc*GNAT to the similarly sized, but catalytically inert, phenylalanine. The Y97F *Lc*GNAT variant also showed a significantly decreased catalytic activity (Fig. 3 ▸). These mutational results strongly suggest that *Lc*GNAT shares its catalytic mechanism with other GNATs, although it remains unclear which tyrosine (Tyr97 or Tyr142) functions as a general acid in the case of *Lc*GNAT and *Bs*PaiA. Speculatively, in GNATs where both tyrosine residues are present one tyrosine could act as an acid while the other might position the acetyl group.

## 4. Data availability

Model coordinates and diffraction data have been deposited with the Protein Data Bank under accession code 8osp. X-ray diffraction images have been deposited at https://doi.org/10.5281/zenodo.7848164.

## Supplementary Material

PDB reference: *Lc*GNAT, 8osp

X-ray diffraction images.: https://doi.org/10.5281/zenodo.7848164

Supplementary Figures. DOI: 10.1107/S2053230X2300571X/us5147sup1.pdf

## Figures and Tables

**Table 1:** X-ray diffraction data statistics and model parameters for *Lc*GNAT

PDB code	8osp
Space group	*P*2_1_
*a*, *b*, *c* (Å)	60.68, 37.08, 83.65
α, β, γ (°)	90, 97.67, 90
Molecules in asymmetric unit	2
X-ray data	
X-ray source	PX1, SLS
Detector	EIGER 16M X
Wavelength (Å)	1.00002
Resolution (Å)	36.48–1.95 (2.00–1.95)
Unique reflections	26391 (1612)
Multiplicity	6.8 (6.5)
Completeness (%)	96.4 (82.3)
〈*I*/σ(*I*)〉	10.03 (2.43)
*R*_merge_(*I*) (%)	15.5 (102.9)
CC_1/2_ (%)	0.996 (0.77)
Refinement	
No. of reflections (work/*R*_free_)	26375/1319
No. of protein residues	349
No. of waters	196
Ligands†	5 × GOL, 1 × PO_4_, 4 × MES
*R* factor/*R*_free_ (%)	18.2/22.1
R.m.s.d.s	
Bond lengths (Å)	0.003
Angles (°)	0.68
Ramachandran plot	
Favoured (%)	98.84
Allowed (%)	1.16
Outliers (%)	0

† PO_4_, phosphate; GOL, glycerol; MES, 2-ethanesulfonic acid.
