# Supplementary material for: Crystal structure of a GCN5-related N-acetyltransferase from Lactobacillus curiae
Source: Acta Crystallogr F Struct Biol Commun. 2023 Aug 9;79(Pt 8):217–23. doi: 10.1107/S2053230X2300571X (PMC10416765; doi:10.1107/S2053230X2300571X)
Supplement: Supplementary file 1 [file f-79-00217-sup1.pdf]

STRUCTURAL BIOLOGY
COMMUNICATIONS

Volume 79 (2023)

Supporting information for article:

Crystal structure of a GCN5-related *N*-acetyltransferase from *Lactobacillus curiae*

Jennifer R. Fleming, Franziskus Hauth, Jörg S. Hartig and Olga Mayans

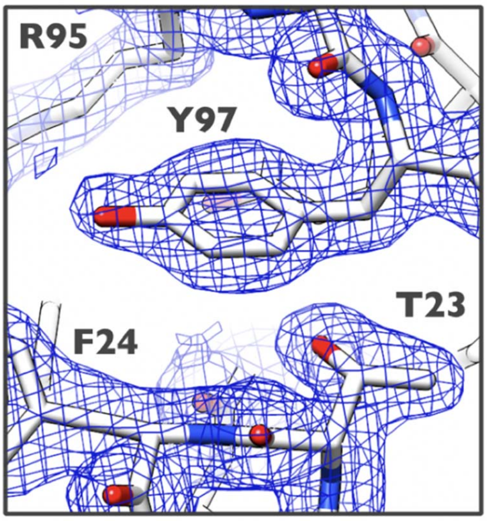

Figure S1 Representative electron density. $(2mF_{obs}\text{-}DF_{calc})\alpha_{calc}$ electron density map contoured at 1σ for the region local to the catalytically relevant residue Y97.

A

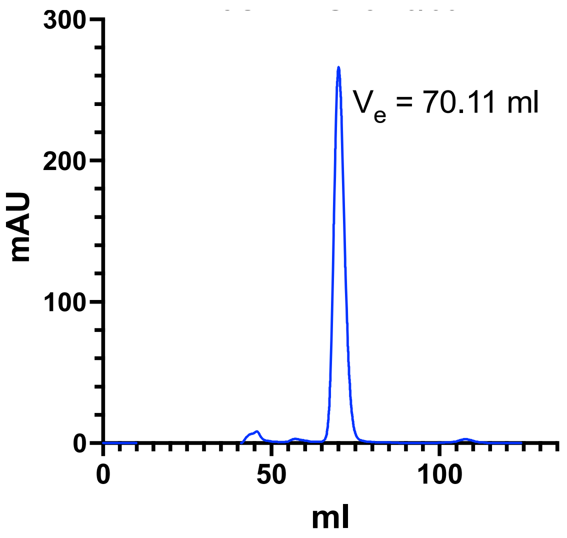

B

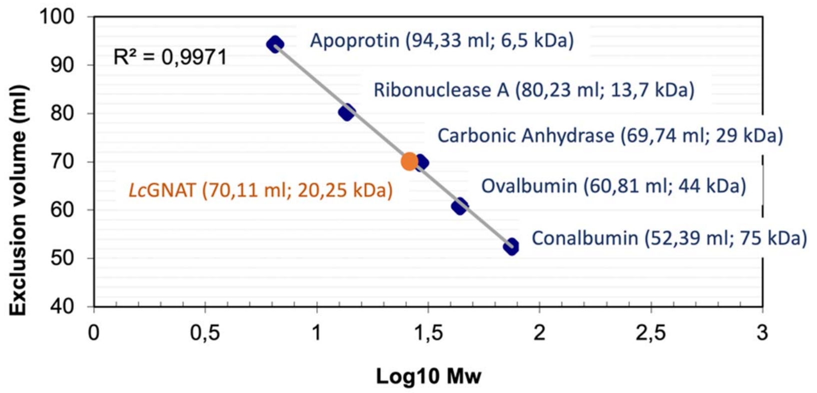

Figure S2 Size exclusion chromatogram of LcGNAT. Size exclusion was performed on a Superdex S75 16/60 column (GE Healthcare) pre-equilibrated in 50 mM Tris pH 8.0, 100 mM NaCl. The loaded sample (volume approx. 3ml) had been concentrated to 6 mg/ml; **B.** The exclusion volume (V_e) of *Lc*GNAT (orange), interpreted in the light of column calibration standards (blue), suggests a monomeric form of the protein (MM= 20.25 kDa; calculated from sequence data). For each protein, exclusion volume and molecular mass are quoted in brackets.

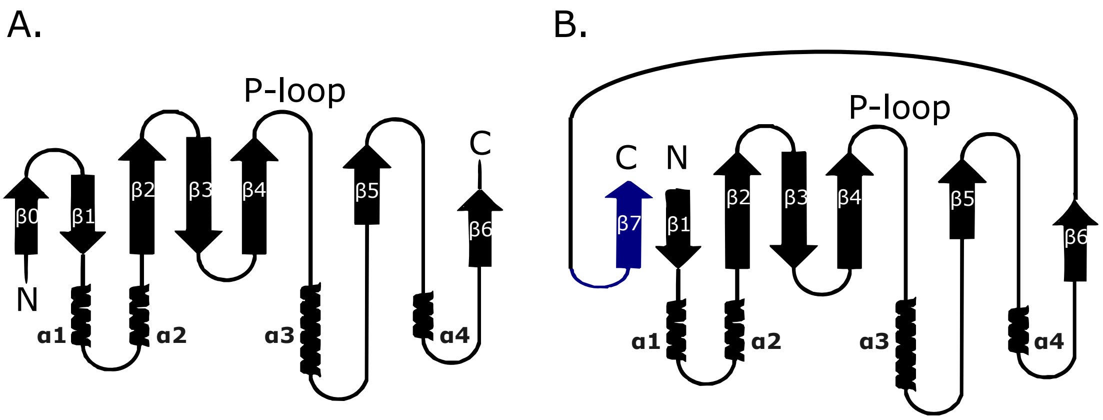

Figure S3 Topological differences in GNAT enzymes. **(A)** Canonical topology of the GNAT superfamily **(B)** and *Lc*GNAT topology representative of the subfamily of polyamine acetyltransferases. In the latter, β-strand β0 is not present. Instead, an extra C-terminal β-strand β7 (dark blue) forms an antiparallel interaction with β-strand β1.
